# Uncovering the dual role of RHAMM as an HA receptor and a regulator of CD44 expression in RHAMM-expressing mesenchymal progenitor cells

**DOI:** 10.3389/fcell.2015.00063

**Published:** 2015-10-15

**Authors:** Mandana Veiseh, Sean J. Leith, Cornelia Tolg, Sallie S. Elhayek, S. Bahram Bahrami, Lisa Collis, Sara Hamilton, James B. McCarthy, Mina J. Bissell, Eva Turley

**Affiliations:** ^1^Life Sciences Division, Lawrence Berkeley National LaboratoriesBerkeley, CA, USA; ^2^Palo Alto Research Center (a Xerox Company)Palo Alto, CA, USA; ^3^Departments of Oncology/Biochemistry/Surgery, Western Schulich School of Medicine, London Regional Cancer Program, Western UniversityLondon, ON, Canada; ^4^Department of Laboratory Medicine and Pathology, Masonic Comprehensive Cancer Center, University of MinnesotaMinneapolis, MN, USA

**Keywords:** CD44, RHAMM, hyaluronan, HMMR, progenitor

## Abstract

The interaction of hyaluronan (HA) with mesenchymal progenitor cells impacts trafficking and fate after tissue colonization during wound repair and these events contribute to diseases such as cancer. How this interaction occurs is poorly understood. Using 10T½ cells as a mesenchymal progenitor model and fluorescent (F-HA) or gold-labeled HA (G-HA) polymers, we studied the role of two HA receptors, RHAMM and CD44, in HA binding and uptake in non-adherent and adherent mesenchymal progenitor (10T½) cells to mimic aspects of cell trafficking and tissue colonization. We show that fluorescent labeled HA (F-HA) binding/uptake was high in non-adherent cells but dropped over time as cells became increasingly adherent. Non-adherent cells displayed both CD44 and RHAMM but only function-blocking anti-RHAMM and not anti-CD44 antibodies significantly reduced F-HA binding/uptake. Adherent cells, which also expressed CD44 and RHAMM, primarily utilized CD44 to bind to F-HA since anti-CD44 but not anti-RHAMM antibodies blocked F-HA uptake. RHAMM overexpression in adherent 10T½ cells led to increased F-HA uptake but this increased binding remained CD44 dependent. Further studies showed that RHAMM-transfection increased CD44 mRNA and protein expression while blocking RHAMM function reduced expression. Collectively, these results suggest that cellular microenvironments in which these receptors function as HA binding proteins differ significantly, and that RHAMM plays at least two roles in F-HA binding by acting as an HA receptor in non-attached cells and by regulating CD44 expression and display in attached cells. Our findings demonstrate adhesion-dependent mechanisms governing HA binding/ uptake that may impact development of new mesenchymal cell-based therapies.

## Introduction

Wound and tumor microenvironments are dynamic composites of in-trafficking and resident cells within a scaffold of remodeling or “provisional” extracellular matrix (Bissell and Hines, 2011; Catalano et al., 2013; Mehner and Radisky, 2013; Bhat and Bissell, 2014; Blonska et al., 2015; Chen et al., 2015). Among the many non-immune cell types, mesenchymal progenitor cells have emerged as critical players in wound repair and diseased tissue (e.g., cancer) microenvironments (Cuiffo and Karnoub, 2012; Vannucci, 2014; Kfoury and Scadden, 2015). Mesenchymal progenitor cells differentiate into activated fibroblasts, myofibroblasts, adipocytes, endothelial, and other cell types that are essential for normal wound repair and are increasingly used as a cell therapy to improve wound repair (Glenn and Whartenby, 2014; Hossain et al., 2014; King et al., 2014; Petrof et al., 2014; Wang et al., 2014a; Zahorec et al., 2015). Mesenchymal cells also contribute to formation of cancer-associated fibroblasts (CAFs) present in the tumor microenvironments that facilitate neoplastic progression (Barcellos-de-Souza et al., 2013; Mehner and Radisky, 2013; Cortez et al., 2014; De Wever et al., 2014; Xiong et al., 2015). CAF transcriptomes resemble those of wounded or activated fibroblasts, and both wound fibroblast and CAF signatures have prognostic value in a number of cancers (Casey et al., 2009; Lim et al., 2011; Tchou et al., 2012; Herrera et al., 2013; Mehner and Radisky, 2013; Paulsson and Micke, 2014; Isella et al., 2015). Part of the function of mesenchymal progenitor cells in wounds and diseased tissues are the production and metabolism of key extracellular matrix components.

The polysaccharide hyaluronan (HA) is an ECM molecules produced by mesenchymal progenitor cells that performs key functions in wounds and diseases such as cancer (Itano et al., 2008; Veiseh and Turley, 2011; Nikitovic et al., 2013a; Provenzano and Hingorani, 2013; Bourguignon et al., 2014; Dicker et al., 2014; Kouvidi et al., 2014; Schmaus et al., 2014; Tolg et al., 2014; Briggs et al., 2015; Finlayson, 2015; Misra et al., 2015; Nagy et al., 2015). Like many ECM molecules HA is fragmented in wound and tumor microenvironments as a result of free radical and enzymatic (hyaluronidase) de-polymerizing activity (Stern and Maibach, 2008; Heldin et al., 2013; Nikitovic et al., 2013a,b; McAtee et al., 2014; Parsons, 2015). HA fragments have different signaling properties than the native HA polymer as a result of their selective binding to HA receptors. During both wound repair and neoplastic progression, HA fragments appear to be more active in promoting in-trafficking of immune and stem cells than native HA (>500 kDa) (Jiang et al., 2011; Petrey and de la Motte, 2014; Schaefer, 2014; Singleton, 2014; Tolg et al., 2014; Ghosh et al., 2015). In wounds, both native HA and HA fragments interact with mesenchymal progenitors and contribute to their fate determination (Khaldoyanidi et al., 2014; Kota et al., 2014; Kouvidi et al., 2014). HA is constitutively elevated in many cancers and its increased accumulation in either the tumor or peri-tumor stroma is associated with poor outcome in a number of malignancies in particular breast and prostate cancers (Toole, 2004; Tammi et al., 2008; Heldin et al., 2013; Khaldoyanidi et al., 2014; Kouvidi et al., 2014). Mesenchymal progenitor cells are emerging as key regulators of HA metabolism during progression of these cancers (Astachov et al., 2011; Caralla et al., 2012; Khaldoyanidi et al., 2014; Kouvidi et al., 2014). However, the expression of HA receptors and/or their respective roles in binding and uptake of this polysaccharide by mesenchymal progenitor cells are not yet well characterized.

The current studies were designed to identify the key HA receptors that mediate HA interaction and uptake by mesenchymal progenitor cells. We utilize 10T½ cells as a model of mesenchymal progenitor cells since these have been shown to exhibit the capacity to differentiate into adipocytes, chondrocytes, myofibroblasts, smooth muscle cells, and endothelial cells (Salvatori et al., 1995; Artaza et al., 2008; Kennard et al., 2008; Wang et al., 2010). We show that these cells utilize two well characterized HA receptors CD44 and RHAMM/HMMR for HA binding and uptake. Importantly, receptor usage for HA internalization is cell attachment dependent. Whereas adherent 10T½ cells use CD44 as an endocytic HA receptor, suspended and newly attached cells utilize RHAMM/HMMR as the dominant HA receptor.

## Methods

### Preparation of F-HA and G-HA probes

Texas Red, Cy5.5, or gold was conjugated to HA (240 kDa, Hyal pharmaceutical), and according to previously described methods for use in adherent cells (Collis et al., 1998; Gouin and Winnik, 2001; Veiseh and Turley, 2011). For analysis of suspended cells, Alexa fluor 647-HA probes were developed by conjugating polydisperse 240 kDa Sodium Hyaluronate (1%, Lifecore) to Alexa-fluor 647-hydrazide dye (120 kDa, Life Technologies). Two-hundred μL of HA and 300 μL dye (1 mg/mL) were added to 1 mL of conjugation buffer [20 mM MES, 30% EtOH, 0.0028 g/mL 1-Ethyl-3-(3-dimethylaminopropyl) carbodiimide]. The mixture was set on a rocker at room temperature in the dark overnight. The solution was then dialyzed against 1 × PBS in 3 mL slide-a-lyzer cassettes (10,000 mWCO, Thermoscientific), changing the dialysate every 24 h for 4 days. For transmission electron microscopy (Maxwell et al., 2011), gold was linked to HA (240 KDa, G-HA) using previously published protocols for preparing gadolinium-HA (Gouin and Winnik, 2001).

### Addition of HA-probes to adherent cells

Medium of 40% subconfluent cell cultures was changed to serum-free low glucose DMEM (Gibco, BRL) containing 4 μg/ml transferrin (Gibco, BRL), 4 μg/ml insulin (Gibco, BRL), and 10 mM Hepes (Sigma). After overnight incubation, cells were exposed to 100 μg/ml Texas red, Cy5.5, and or gold-HA for 10 min in defined (low glucose DMEM) medium containing insulin and transferrin. 100 ug/ml FITC-dextran (10 KDa, Molecular Probes) was added at the same time to provide a process for standardizing fluorescent intensity and as a measure of constitutive uptake. For confocal microscopy, cells were grown on coverslips and after F-HA uptake were rinsed twice in cold 5X PBS and fixed in 2% freshly prepared paraformaldehyde + 1% cetylpyridinium chloride as described. Coverslips were then washed in 1 × PBS and mounted using fluorescent mounting medium (DAKO).

### Tissue fixation for transmission electron microscopy

For transmission electron microscopy, cells were fixed with 2.5% glutaraldehyde, 1.5% paraformaldehyde, and 2.5% DMSO in cacodylate buffer, then post fixed in osmium tetroxide (Veiseh et al., 2014).

### HA fragment size analyses

For HA fragment uptake analyses, Texas red end labeled HA fragments (HA4, HA8, HA12, HA26, and HA30, kind gift of M. and R. Tammi, U. Kuopio, Finland) were added to cells, which were fixed and processed as above. HA fragments were generated by digestion with hyaluronidase and purified by chromatography as described (Siiskonen et al., 2013).

### Quantification of binding and uptake of F-HA in attached cells

Cells were examined with a Zeiss Axiophot 100 confocal microscope and z-axis images taken through the middle of cells were analyzed for Texas red or Alexa647-HA, collectively termed F-HA, uptake. UTHSCA image Tool (version 1.28, University of Texas Health Sciences Center in San Antonio) was used and zero thresholding of equal central sections taken from the perinuclear areas were implemented as represented in **Figure 2A**. Staining of cells with different cell thicknesses was standardized using a ratio of Texas red-HA:FITC-dextran fluorescence. Background staining, which included autofluorescence and fluorescence following uptake of Streptomyces hyaluronidase-digested Texas red HA (digested to completion), was subtracted from the raw data.

### Flow cytometry of suspended cells

10T½ (parental) and RHAMM-transfected 10T½ cells (RHAMM-10T½) were brought to 50% confluence in low-glucose DMEM media (10% FBS) and released from the culture surface using 1 × non-enzymatic dissociation solution (Sigma-Aldrich). Cells were pelleted then re-suspended at 2.5 × 10^5^ cells per 100 μL. For non-blocking conditions, cells were then incubated with 1:20 monoclonal mouse anti-RHAMM (6B7B7) for 30 min on ice. Cells were spun and washed twice with 1 mL 1 × sterile PBS. They were then re-suspended in 1 mL PBS and incubated with Rat anti-mouse Alexa fluor 488 (1:2000) secondary antibody, together with Rabbit anti-mouse CD44-RPE conjugate (1:1000) (Clone IM7.8.1, Life Technologies) and Alex-fluor 647-HA (133 μg/mL) for 30 min. Cells were then washed and spun three times with 1 mL 1 × sterile PBS for flow cytometry analysis.

Cell fluorescence was detected using a FACSCalibur II flow cytometry machine and data were acquired using the Cellquest™ Pro analysis program (BD Biosciences). Forward Scatter and side scatter light was collected through a 488/10 filter using the blue (488 nm) laser, with side scatter collected at a 90° angle from the original light path. RHAMM-bound Alexa fluor 488 and CD44-PE fluorophores were excited using the 488 nm blue laser, with the excitation wavelengths being detected by the FL1 (530-30) and FL2 (585/42) light filters, respectively. F-HA was excited by the 635 nm red diode laser, and detected by the FL4 (661/16) light filter. The fluorescence voltage levels were adjusted using the unstained and non-immune IgG control cells, moving the events to the bottom left corner of the dot plot. Using single- and double-stained controls, each fluorescent marker was adjusted by compensation to its appropriate location (not appearing in an improper filter). Twenty thousand gated events were acquired per sample for data analysis. Flow data was then analyzed using Flowjo V10 (Treestar Inc.).

### Blocking HA receptors and inhibiting HA production in suspended cells

For assessing the consequences of blocking HA receptors on F-HA binding and CD44 display, suspended cells were prepared as described above then exposed to blocking antibodies prior to F-HA or CD44 display analyses for 1 h in cell culture medium 37°C. Cells were then prepared for flow cytometry by incubation with rabbit anti-mouse CD44-RPE antibody (Life technologies) or F-HA as in five above. The CD44 function blocking antibody (KM201) has previously been shown to reduce HA:CD44 interactions (Culty et al., 1990) and the RHAMM function blocking antibody (exon 8 rabbit polyclonal antibody) reduced migration of RHAMM-rescued but not RHAMM–/– cells (Tolg et al., 2006).

To assess CD44 membrane display when endogenous HA production has been blocked, under (4-MU) treatment, adherent RHAMM-transfected 10T½ cells were incubated with 1 mM 4-Methylumbelliferone (4-MU) for 4 h at 37°C (Rilla et al., 2004). Cells were then prepared for flow cytometry as previously described above and stained using the Rabbit anti-mouse CD44-RPE antibody (Life Technologies).

### RHAMM transfection of 10T½ fibroblasts

10T½ fibroblasts were stably transfected with an N-terminal truncated RHAMM cDNA(Δ163aa)/hygromysin construct driven by a ß-actin promoter (Hall et al., 1995). This RHAMM cDNA encodes an isoform commonly expressed in cancer cell lines (Savani et al., 1995; Tolg et al., 2006; Hamilton et al., 2007) and following tissue wounding. Stably transfected cells were selected in G418 and cloned (Hall et al., 1995). The clone (designated RHAMM-10T½) was used in the present study.

### Western blots and HA-sepharose pull-down assays

Western blot analysis of RHAMM expression was performed as previously described (Hamilton et al., 2007) using polyclonal anti-peptide antibodies screened for specificity by RHAMM−∕− and RHAMM+∕+ fibroblast lysates. CD44 western blot analysis on 10T½ fibroblasts as well as RHAMM-transfected 10T½ cells was performed as previously described (Tolg et al., 2006), with the exception of using primary anti-CD44 antibodies (AF6127, R&D systems) at 1 μg/mL overnight. Secondary antibodies were incubated for 1 h at room temperature (Bethyl Laboratories). Flow cytometry was performed using anti-CD44 antibodies (IM7, Pharmingen). HA-sepharose pulldowns were performed as described previously (Tolg et al., 2012). For antibody blocking experiments, antibody was added to cultures for 30 min followed by Texas red-HA as described above.

### Microarray analysis and QPCR

Microarray analysis and QPCR were performed as previously described (Tolg et al., 2012). In brief, subconfluent 10T½ and RHAMM-transfected 10T½ cells were cultured overnight in serum free defined medium (DMEM, 4 μg/mL insulin, 8 μg/mL transferrin). Total RNA from three biological replicates was isolated using TRIzol Reagent (Invitrogen) and purified using the QIAGEN RNAeasy kit (Qiagen, Valencia, CA) following the manufacturer's instructions. RNA quality was assessed using Agilent 2100 Bioanalyzer (Agilent Technologies, Santa Clara, CA) and the RNA 6000 Nano kit (Caliper Life Sciences, Hopkinton, MA). Double-stranded cDNA was synthesized using SuperScript II (Invitrogen) and oligo primers. Labeled cRNA was prepared using the BioArray High-Yield RNA Transcript Labeling kit (Enzo Biochem, New York, NY) and Mouse Genome 430 2.0 GeneChips (Affymetrix, Santa Clara, CA) were hybridized with 10 μg labeled cRNA as described in Affymetrix GeneChip Technical Analysis manual (Affymetrix, Santa Clara, CA). Biotinylated cRNA was detected with streptavidin-phycoerythrin (S-P) using a GeneChip Fluidics Station 450. GeneChips were scanned with the Affymetrix GeneChip Scanner 3000 (Affymetrix). Signal Intensities for all genes were generated using GCOS1.3 (Affymetrix) using default values for the Statistical Expression algorithm parameters and a target signal of 150 for probe sets and a normalization value of 1. Data were then transformed (< 0.01 set to 0.01) and normalized per chip to the 50th percentile and per gene to controls. To determine the effect of RHAMM on gene expression, RHAMM-transfected 10T½cells were compared to10T½cells. GeneSpring GX 7.3.1. (Agilent Technologies) was used to identify fold changes in gene expression between the two cell lines by applying a *t*-test with Bonferroni multiple testing correction with a significance cut-off of 0.05. Genes with a two-fold or higher difference in expression were considered for further analysis. All gene chips were processed at the London Regional Genomics Centre (Robarts Research Institute, London, ON, Canada). Signaling pathways were identified by Ingenuity Pathway Analysis (Ingenuity Systems, Redwood City, CA).

### Cytoskeleton disruption with cytochalasin B

Cytochalasin B (10 μM, Sigma) was added to adherent RHAMM-transfected fibroblasts for 30 min prior to the addition of Texas-red HA.

### Statistical analyses

Statistical significance of experiments comparing means of fluorescence intensity (F-HA uptake) was calculated using 2-tailed 2 sample *t*-tests. The means compared were: unblocked and short HA fragment-blocked cells (**Figure 2C**) 10T½cells and HA-mutant cells (**Figure 7C**), 10T½MEFs, and RHAMM-transfected 10T½cells (**Figures 3A,C,D**, **4C**, **5A**), and cells blocked with an IgG or anti-RHAMM antibody (**Figure 5A**). Minimum significance was set at *p* < 0.05.

## Results

### Suspended and attached 10T½ cells bind and internalize F-HA and G-HA

**Figure 1:**
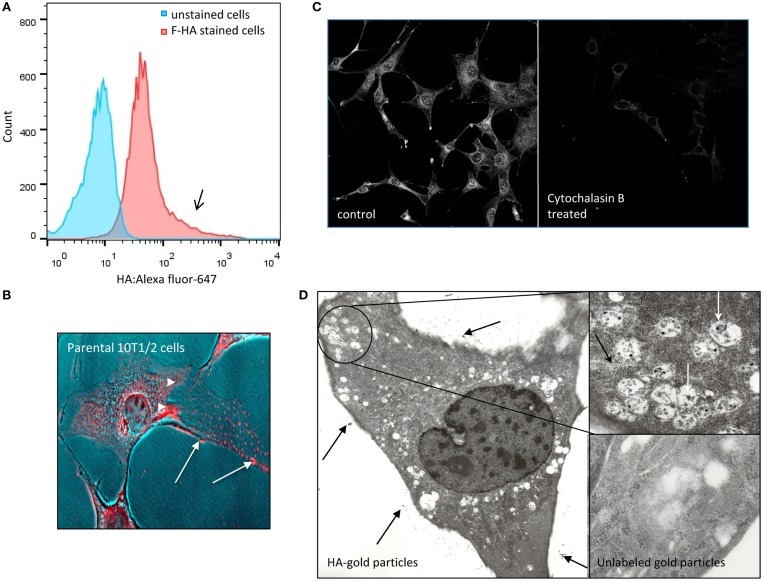
**F-HA binds to and internalized by detached and adherent 10T½ cells**. **(A)** Flow cytometry analysis shows heterogeneous binding (high binding notated by black arrow) and uptake of F-HA by non-adherent parental 10T½ cells (red). Cells that were not exposed to F-HA (e.g., unstained cells) are shown as a negative control (blue). **(B)** Confocal micrograph of F-HA internalized by adherent 10T½ cells shows the probe is located at the cell surface (arrows), as well as inside the cells where it accumulates in the perinuclear and nuclear areas (arrowheads). **(C)** F-HA uptake in adherent RHAMM-10T½ cells is blocked by disruption of the actin cytoskeleton using cytochalasin B confirming a role for the cytoskeleton in F-HA uptake by adherent cells. **(D)** Transmission electron micrograph confirms that G-HA accumulates at the extracellular face or the glycocalyx of cells (arrows) and is internalized in vesicles (inset), which are abundant in cell processes, and in the peri-nuclear areas, and are associated with the cytoskeleton (black arrow, inset) consistent with a role for endocytic processes in internalization of the HA probe.

**Figure 2:**
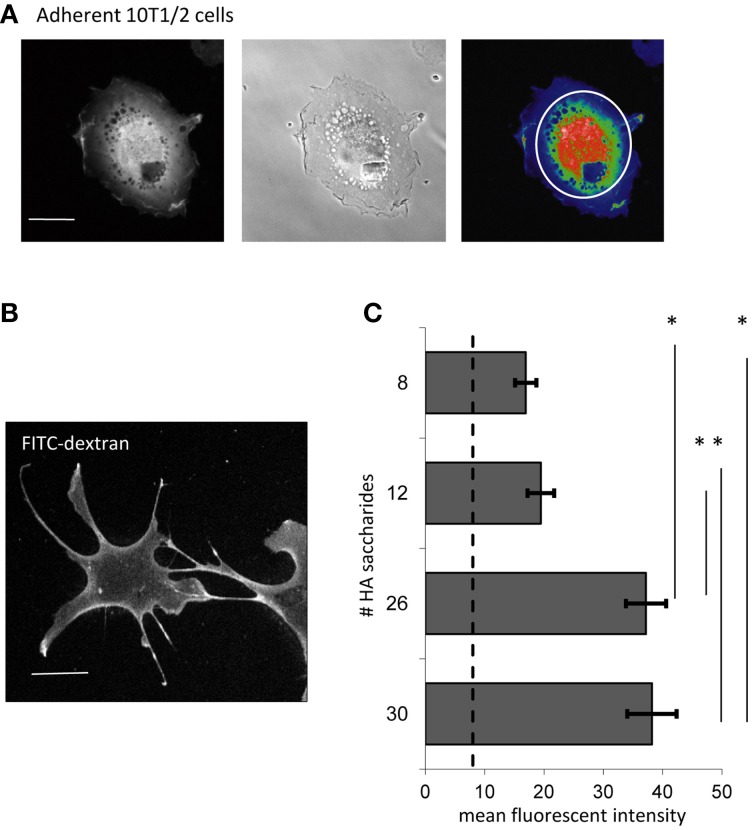
**F-HA oligosaccharides are internalized by 10T½ cells**. **(A)** Confocal micrograph showing the perinuclear and nuclear area used for quantification of texas red-HA in adherent cells (left image); middle micrograph is a phase contrast image of the cell and right image is a heat map of the fluorescent texas red-HA staining. **(B)** Confocal micrograph of adherent 10T½ cells shows the fluorescent uptake of FITC-dextran, which is not HA receptor mediated. **(C)** Internalization of sized HA fragments end-labeled with Texas red, was measured against a background of FITC-dextran uptake. Results show that HA polymers of 8–12 saccharides are internalized slightly above the FITC-dextran background, but internalization is significantly increased when polymer sizes reach to 26 or more saccharides. Confocal micrographs are representative images (Bar = 10 μm). Values are the mean and SEM of *n* = 40 cells. Asterisks indicate statistical significance (*p* < 0.05).

To begin to characterize 10T½ mesenchymal progenitors for their ability to metabolize HA, cells were exposed to Alexa-647- or Texas Red-HA (collectively termed F-HA) (Figures 1A–C), and Gold-HA (G-HA) probes (Figure 1D). Bound probes were detected using flow cytometry (Figure 1A), confocal (F-HA, Figure 1B) or transmission electron microscopy (G-HA, Figure 1D). Flow cytometry shows that suspended 10T½ cells bind F-HA in a heterogeneous manner as indicated by tailing of the binding profile (Figure 1A, arrow). Confocal analyses (e.g., Figure 1B) of adherent 10T½ cells confirm that the F-HA binds to cell surfaces (e.g., arrows, Figure 1B) and is internalized in cytoplasmic vesicles that are associated with the cytoskeleton (Figure 1B, arrowheads). The importance of the actin cytoskeleton to internalization of F-HA is further demonstrated by the ability of cytocholasin B, which disrupts actin filament assembly, to inhibit F-HA uptake (Figure 1C). F-HA also accumulates in the perinuclear area and is apparent in the nuclei of adherent cells (Figures 1B, 2A, heat map circle). This vesicular uptake pattern is confirmed by TEM using gold labeled HA (G-HA) and unlabeled gold as a negative control (Figure 1D). Analysis of cell sections confirm that G-HA is present in a pericellular coat (Siiskonen et al., 2015) (Figure 1D, black arrows) and in cytoplasmic vesicles (Figure 1D, inset, white arrows) that are present in cell processes and in the perinuclear area. By contrast, uptake of FITC-dextran, used as a marker for HA receptor independent uptake (pinocytosis), shows low to no accumulation in the perinuclear/nuclear regions (compare Figures 2A,B). The presence of labeled HA within vesicles is consistent with an HA receptor mediated endocytic mechanism (Thankamony and Knudson, 2006).

### F-HA probe uptake is polymer size and cell attachment dependent

The binding of HA to its receptors is typically size dependent while non-specific uptake (e.g., pinocytosis) is not (Mills and Finlay, 1994; Ma et al., 2013). We therefore first evaluated the binding and uptake of sized HA oligosaccharides in adherent 10T½ cells and compared results with uptake of FITC-dextran, used as a marker for non-HA receptor mediated internalization (Figures 2A–C). Since RHAMM and CD44 are expressed on mesenchymal progenitor cells (Shigeishi et al., 2013), HA oligosaccharides ranging from 8 to 30 saccharides, which are known to bind to these receptors (Nikitovic et al., 2013a), were analyzed for uptake (Figure 2C). Notably, CD44 binds to a minimum of six dissacharides but is only clustered by larger polymers, which is required for endocytosis (Yang et al., 2012). 10T½ cells internalize F-HA_8_ to F-HA_12_ at a level slightly above the FITC-dextran background (Figure 2C, dotted line) but F-HA_26−30_ are internalized to a significantly greater extent than these two smaller HA fragments. These results show that F-HA internalization is influenced by F-HA size in a manner that is consistent with a role for HA receptors in the internalization process.

**Figure 3:**
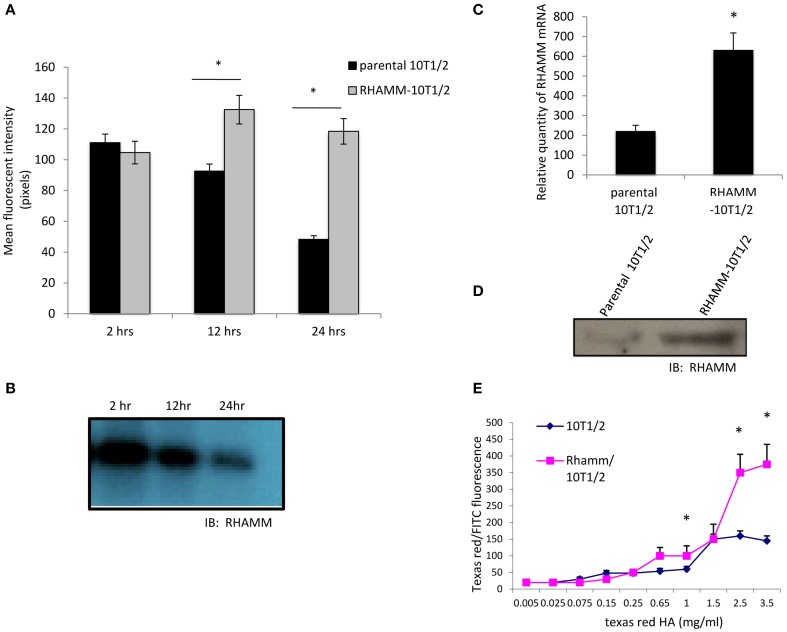
**RHAMM overexpression in 10T½ cells increases F-HA uptake**. **(A)** F-HA uptake is highest in newly plated 10T½ cells but drops over time as cells become firmly attached and form an organized actin cytoskeleton (e.g., 24 h time point). In contrast, F-HA uptake in RHAMM-10T½ cells remains high between 2 and 24 h. **(B)** Western blot shows RHAMM protein expressed by parental 10T½ cells decreases with time after subculture. **(C)** Q-PCR analyses confirm that RHAMM mRNA expression is significantly higher in RHAMM-10T½ cells than in the parental counterpart at 24 h after subculture (asterisk indicates statistical significance, *p* < 0.05). **(D)** Western blot shows that RHAMM protein is also expressed at higher levels in RHAMM-10T½ cells vs. parental cells at 24 h after subculture. **(E)** Analysis of F-HA binding to RHAMM-10T½ and parental cells with increasing F-HA concentration. Graphs shows that RHAMM-10T½ cells bind more F-HA than parental counterparts suggesting RHAMM transfection increases display of HA receptors. Values are the mean and SEM of *n* = 50 cells. Asterisks indicate statistical significance (*p* < 0.05).

Here, we show that F-HA uptake is also affected by the adhesion status of 10T½ cells. Uptake is highest in 10T½ cells 2 h after plating when cells are initially attaching, but drops significantly between 12 and 24 h after plating (Figure 3A, black bars), when cells are firmly adherent as judged by a flattened morphology. Constitutive RHAMM expression is rare in most tissues or confluent cultures (Savani et al., 1995; Tolg et al., 2006) and has been reported to drop with time following cell plating (Zhang et al., 1998). Our results confirm this transient expression in 10T½ cells between 2 and 24 h post-subculture (Figure 3B). A RHAMM cDNA was therefore transfected into 10T½ cells to sustain expression of this protein and to determine if this forced expression stabilizes high F-HA uptake after cell subculture. A more than two-fold increase in the expression of RHAMM is confirmed with mRNA microarray (Table 1), Q-PCR (Table 1 and Figure 3C) and western blot (Figure 3D) assays, measuring expression in adherent cells 24 h after subculture. RHAMM overexpression stabilizes F-HA binding/internalization in 10T½ cells over time after subculture so that even at 24 h levels remain high (Figure 3A, gray bars). Uptake of increasing F-HA concentrations by parental and RHAMM-10T½ cells at 24 h after subculture was next quantified to determine if increased F-HA uptake in RHAMM-10T½ results from increased HA receptor display. For these experiments, F-HA uptake was standardized against that of FITC-dextran internalization to account for the role of pinocytosis in probe uptake, which is not HA receptor mediated. F-HA uptake by the parental 10T½ cells reaches saturation between 1.0 and 1.5 mg/ml F-HA but continues to increase in RHAMM-transfected cells to 3.5 mg/ml (Figure 3E). These results suggest that sustained expression of RHAMM increases F-HA uptake through increasing HA receptor expression/display, which is required for F-HA internalization.

**Table 1:** **Microarray analyses of HA receptor expression changes in RHAMM-transfected vs. parental cells**.

**Gene**	**Fold change**		***P*****-value**	
	**Microarray**	**Q-PCR**	**Microarray**	**Q-PCR**
RHAMM/HMMR	2.37^*^	2.95^*^	2.369E-08	0.05
CD44	2.29^*^	2.69^*^	2.93E-07	0.05
STAB2	1.75	ND	1.70E-05	ND
LYVE1	No change			
STAB1	No change			
KIAA1199 (CEMIP)	No change			
C1QBP (HABP1)	No change			

RHAMM transfection sustains RHAMM mRNA expression and also increases mRNA expression of CD44 but not other characterized HA receptors. (

* p < 0.05).

ND means Not Determined.

### CD44 mediates F-HA uptake in adherent 10T½ cells

**Figure 4:**
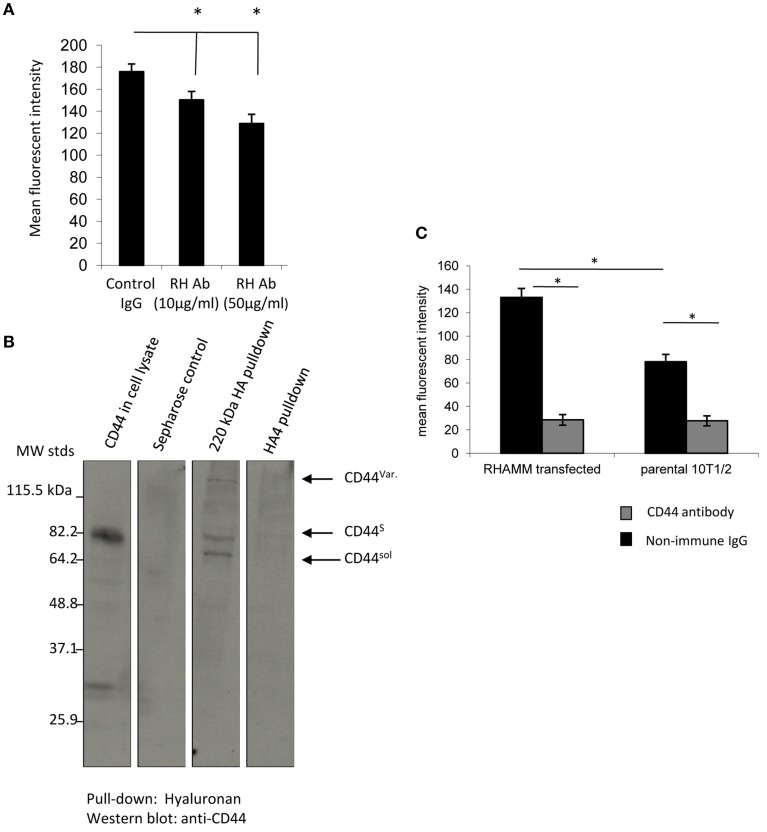
**CD44 binds to F-HA, and the uptake in adherent RHAMM transfected cells is CD44-dependent**. **(A)** F-HA probe uptake in adherent RHAMM-10T½ cells is significantly blocked. Values are the mean and SEM of *n* = 50 cells and asterisks indicate statistical significance (*p* < 0.05) by function blocking anti-RHAMM antibodies but the effect is slight. **(B)** 240 kDa HA-sepharose pulls down CD44 standard, variant and soluble forms from 10T½ cell lysates, but in contrast, and as expected, HA_4_ does not. **(C)** Anti-CD44 antibodies strongly block F-HA uptake in both parental and RHAMM-10T½ cells indicating that this receptor is primarily responsible for the RHAMM-mediated increase in F-HA internalization, and is the major endocytic HA receptor in adherent cells. Values are the mean and SEM of *n* = 50 cells. Asterisks indicate statistical significance (*p* < 0.00001).

Since RHAMM-10T½ cells constitutively express elevated RHAMM, the role of this HA receptor in F-HA internalization by adherent cells was examined. The function blocking anti-RHAMM antibody significantly reduces F-HA uptake by RHAMM-10T½ cells but the effect is slight and does not account for the majority of uptake (Figure 4A). To verify that RHAMM-10T½ cells express HA binding CD44 proteins, pull-down assays using HA-Sepharose were performed (Figure 4B). Results show that CD44 protein is strongly expressed in RHAMM-10T½ cells and that the standard and variant forms of CD44 bind to HA-sepharose. A smaller CD44 protein form (60 kDa), which is likely to be the soluble form of this HA receptor, also binds to HA-Sepharose. The effect of blocking CD44:HA interactions on F-HA uptake by RHAMM-10T½ cells was therefore next examined using a function blocking CD44 antibody (Figure 4C). Results show that this antibody blocked the majority of F-HA uptake in adherent parental and RHAMM-10T½ cells suggesting that CD44 is the primary endocytic HA receptor in both 10T½ cell types. Collectively, these results also predict that sustained RHAMM expression may be affecting expression and/or display of CD44.

### RHAMM transfection regulates CD44 expression in 10T½ cells

**Figure 5:**
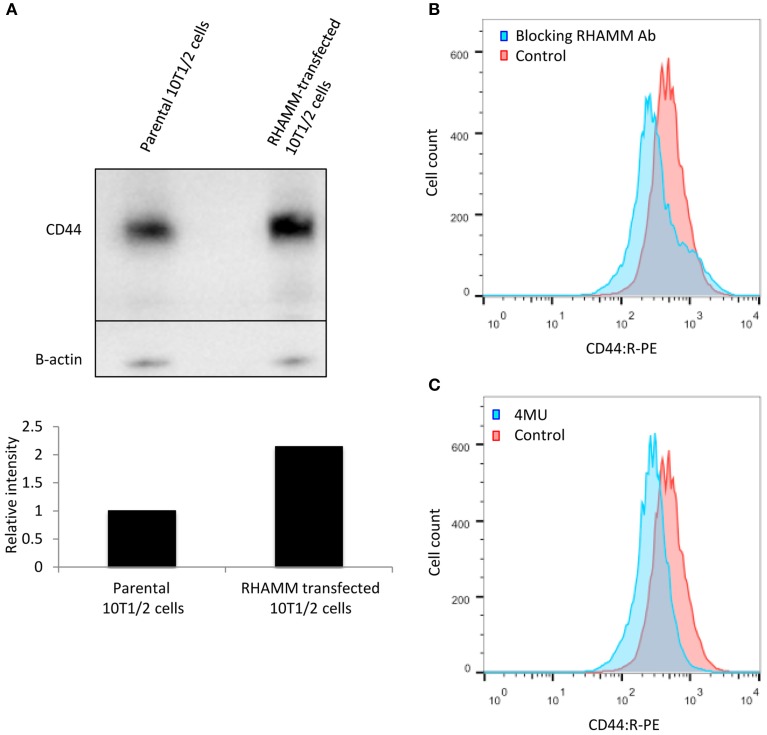
**RHAMM affects CD44 expression**. **(A)** Western blot analysis of adherent RHAMM-10T½ cell lysates reveals an approximately two-fold increase in the expression of CD44s protein compared to the parental cells (replicated twice). **(B)** Function blocking anti-RHAMM antibody reduces the display of CD44 on RHAMM-10T½ cells. **(C)** Reduction of CD44 display is observed when HA production by these cells is inhibited with 4MU.

To assess if RHAMM transfection increases transcription of CD44, RHAMM-10T½ vs. parental 10T½ mRNA microarray analyses were performed. Results confirm that RHAMM expression is significantly increased, as expected and show that CD44 mRNA expression is also increased (Table 1). This effect is selective for CD44 since changes in expression levels of other characterized HA receptors are either not detected or are decreased (STAB2 mRNA levels are decreased). Q-PCR confirms the increase in CD44 mRNA detected by microarray analyses (Table 1) and western blots also demonstrate a similar increase in CD44 protein levels (Figure 5A). These results suggest that RHAMM transfection either promotes CD44 transcription or regulates the stability of the CD44 mRNA. A potential transcriptional effect could be a direct result of RHAMM since it has recently been shown to participate in gene transcription (Meier et al., 2014). Alternatively it could be an indirect effect resulting from its ability to promote signaling pathways that control CD44 expression or mRNA stability. For example, RHAMM could alter signaling through growth factors such as PDGF (Nikitovic et al., 2013a) or ligands such as HA (Tolg et al., 2014). To begin to assess this, the signaling functions of cell surface RHAMM were blocked in RHAMM-10T½ cells using the function-blocking antibody. As well, HA production was inhibited by 4MU and the consequences of these two treatments to CD44 expression was quantified using flow cytometry (Figures 5B,C). The RHAMM antibody reduces CD44 display by approximately 10-fold although a subpopulation of CD44 is unaffected, as indicated by an increased peak width around 10^3^ signal (Figure 5B). Inhibiting HA production by 4MU also reduces CD44 display (Figure 5C). Collectively these results predict that RHAMM signaling, possibly through HA, affects CD44 expression/display of RHAMM-10T½ cells indirectly controlling CD44 mediated HA uptake (Tolg et al., 2012; Bourguignon et al., 2014).

### RHAMM mediates F-HA binding in suspended RHAMM-10T½ cells

The ability of stem cells to traffic to sites of tissue injury via the vasculature requires their survival as non-adherent cells. The ability to survive as suspended cells has been linked to HA:cell interactions amongst other microenvironmental factors that influence susceptibility to apoptosis (Zhang et al., 2015). We therefore analyzed the mechanisms by which suspended RHAMM-10T½ cells bind to F-HA. Multiplexed flow cytometry shows that suspended RHAMM-10T½ cells display both

**Figure 6:**
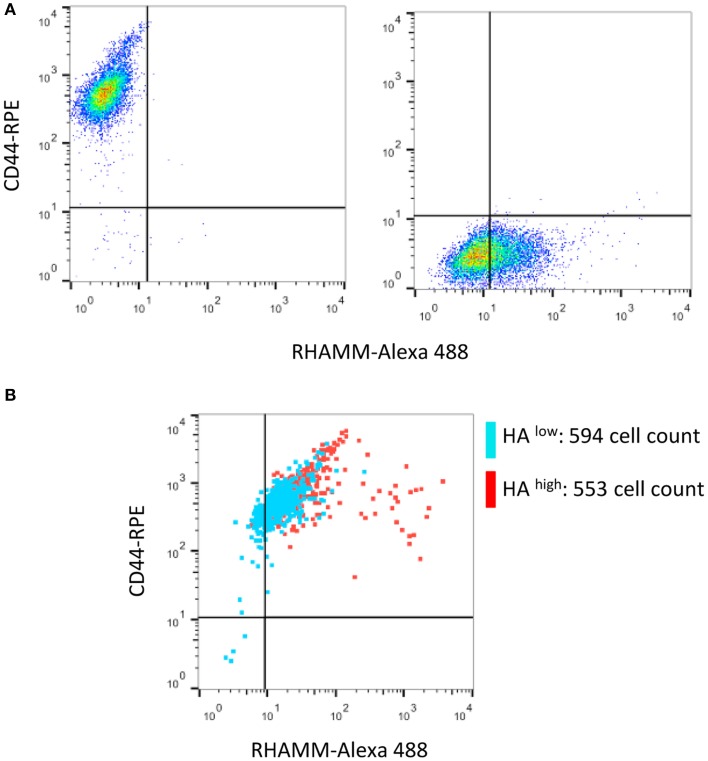
**CD44 and RHAMM are displayed on suspended RHAMM-10T½ cells**. **(A)** Single channel flow cytometry analysis of CD44 and RHAMM levels shows that CD44 levels are higher than RHAMM and that channel bleed through does not occur. **(B)** Multiplexed flow analysis of HA receptor display in RHAMM-10T½ cell subpopulations that bind low (blue, HA^low^ bottom 5% of events) or high (red, HA^high^ top 5% of events) amounts of F-HA probe show that CD44 is abundant in both subpopulations. In contrast, the highest RHAMM display is unique to the HA^high^ subpopulation.

**Figure 7:**
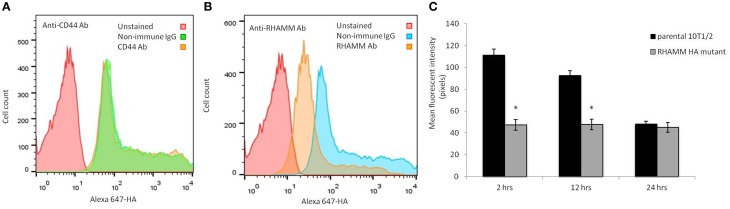
**F-HA probe binding to suspended RHAMM-10T½ cells is reduced by anti-RHAMM but not anti-CD44 antibodies**. **(A,B)** When compared to isotype matched non-immune IgG, function blocking anti-CD44 antibodies do not reduce binding of F-HA probe to suspended 10T½ cells **(A)** whereas anti-RHAMM antibody does **(B)**. **(B, C)** Expression of a dominant negative HA mutant that blocks HA binding to RHAMM significantly reduces F-HA internalization in attaching (2–12 h post subculture) but not firmly adherent 10T½ cells (24 h post subculture). Values are the mean and SEM of *n* = 50 cells. Asterisks indicate statistical significance *p* < 0.05.

CD44 and RHAMM although CD44 is much more abundant than RHAMM (Figure 6A). F-HA binding to these cells is heterogeneous (Figures 6B, 7A,B). Subpopulations that bind the highest levels of F-HA (HA^high^) display higher levels of RHAMM than subpopulations of cells that bind low or no F-HA (HA^low^) (Figure 6B). In contrast, CD44 display is abundant on both HA^high^ and HA^low^ subpopulations. Function blocking anti-CD44 and RHAMM antibodies were used to assess the direct vs. indirect roles of these two HA receptors in binding F-HA to suspended RHAMM-10T½ cells. Unexpectedly, anti-CD44 antibodies do not block F-HA binding to suspended RHAMM-10T½ cells, when compared to the effect of non-immune IgG (Figure 7A). In contrast the anti-RHAMM antibody strongly block F-HA binding in these assays (Figure 7B). However, binding is not completely ablated by blocking RHAMM function, indicating that other receptors are involved in F-HA interactions with the suspended cells. These results prompted us to assess the contribution of RHAMM in F-HA uptake of parental 10T½ cells that are in the process of attaching, a time after subculture when RHAMM expression and F-HA binding/uptake is high (Figure 7C). For these experiments, parental 10T½ cells were transfected with a dominant negative acting RHAMM HA binding mutant that reduces HA binding to this protein. Expression of this mutant ablates the elevated F-HA uptake of cells that are attaching to the growth substratum (Figure 7C, 2–12 h) but not adherent cells (Figure 7C, 24 h). Therefore, the mechanisms used for HA binding/update by suspended or newly attaching are different from adherent 10T½ cells. Collectively, these results suggest that both CD44 and RHAMM can mediate HA binding and uptake via CD44 uptake requires an organized actin cytoskeleton (e.g., Figure 1D) typically found in adherent cells while HA binding via RHAMM predominates in non-adherent cells.

## Discussion

It has been increasingly shown that adherence, fate, and function of mesenchymal stem or progenitor cells are influenced by HA, however less is known about how these cells metabolize HA. Mesenchymal progenitor cells are undifferentiated multi-potent stromal cells originally identified in the bone marrow stroma but which have since been detected in many additional tissues (Hossain et al., 2014; Naderi-Meshkin et al., 2015). These cells traffic to sites of injury where they differentiate into adipocytes, osteoblasts, chondrocytes and fibroblasts/myofibroblasts thus providing a mechanism for self-renewal in the repair of mesenchymal tissues (Ding et al., 2015; Wong et al., 2015). They additionally play key immunomodulatory and nurturing/support roles in stem cell niches required for tissue homeostasis (Glenn and Whartenby, 2014; Kfoury and Scadden, 2015). The homing and multipotential properties of these cells has stimulated interest in a number of clinical applications including tissue engineering for regenerative medicine and delivery tools to treat cancer and other diseases (Ghobadi et al., 2015; Park et al., 2015).

Production and turnover of ECM components (including HA) within repairing and diseased tissues is a key process. Both resident and trafficking msenchymal progenitor cells produce and respond to HA, thus contribute to directing the homing, growth and differentiation of mesenchymal progenitor cells into a number of lineages (Avigdor et al., 2004; Herrera et al., 2007; Astachov et al., 2011; Kishi et al., 2012; Dicker et al., 2014). This differentiation capability of mesenchymal progenitor cells is increasingly utilized in tissue engineering designs (Astachov et al., 2011; Prestwich et al., 2012; Khaldoyanidi et al., 2014). Our major findings are that the 10T½ mesenchymal cell model utilizes both CD44 and RHAMM to bind and internalize HA. Importantly, the cellular contexts in which these receptors function as HA binding proteins differ significantly. CD44 is the primary HA endocytic receptor in adherent mesenchymal cells, however this function requires an organized cytoskeleton typical of adherent cells. By contrast, RHAMM performs a number of functions in HA binding/internalization by these mesenchymal progenitor cells because it both regulates expression of CD44 when cells are adherent and acts as a major HA binding receptor, when 10T½ cells are suspended or newly attached.

Previous reports have extensively documented that CD44 interacts both directly and indirectly with the actin cytoskeleton in adherent cells. Several reports have noted that CD44-mediated HA binding requires an association with phosphorylated ERM (ezrin, radixin, and moesin) proteins and the actin cytoskeleton (Bourguignon et al., 2013). Consistent with our present observations, a previous study showed that disruption of polymerized actin inhibits CD44 clustering and abolishes HA binding in myeloid cells (Brown et al., 2005). Flow cytometry requires the use of suspended cells and thus these predict that HA dependent non-adherent trafficking of mesenchymal cells likely depends more on cell surface RHAMM: HA interactions than CD44: HA interactions.

RHAMM is a multifunctional cytoplasmic protein that, like many unconventionally exported cytoplasmic proteins, appears on the cell surface under very specific conditions of cell stress (Maxwell et al., 2008; Radisky et al., 2009). Inside the cell it binds to such proteins as tubulin, ERK1 and TPX2 that impact mitotic spindle orientation and integrity (Maxwell et al., 2008; Kouvidi et al., 2014; Tolg et al., 2014). Since RHAMM is not an integral membrane protein, it affects activation of HA stimulated signaling pathways by functioning as a co-receptor for integral membrane proteins such as CD44 and/or growth factor receptors (Shigeishi et al., 2014; Tolg et al., 2014). Our data suggest that cell surface RHAMM may not partner with CD44 in the binding and internalization of HA by the non-adherent cells. Instead, RHAMM may partner with other integral membrane receptors such as growth factor receptors (PDGFR, EGFR, or RON) (Nikitovic et al., 2013a; Tolg et al., 2014) or other HA receptors (Forteza et al., 2012; Heldin et al., 2013).

Intracellular RHAMM traffics to the cell nucleus and more recent studies show that it binds to and participates in E2F1 transcriptional functions including expression of fibronectin, providing a context in which intracellular RHAMM regulates gene transcription (Meier et al., 2014). We and others (Gouëffic et al., 2006; Hatano et al., 2011; Foley et al., 2012; Park et al., 2012; Tolg et al., 2014; Wang et al., 2014b) have shown that RHAMM controls activation kinetics and subcellular localization of ERK1,2, MAP kinases that have been reported to promote CD44 expression (Recio and Merlino, 2003; Judd et al., 2012). Cell surface RHAMM can activate ERK1,2 via binding to HA while intracellular RHAMM traffics into the cell nucleus, where it could potentially participate in transcription directly. Defining whether or not signaling that increases CD44 expression originates from cell surface RHAMM-mediated activation of ERK1,2 and/or from direct intracellular RHAMM transcriptional function requires further study.

Our findings are relevant to both tissue repair and to such diseases as cancer (Schäfer and Werner, 2008; Astachov et al., 2011; Kishi et al., 2012; Tolg et al., 2014; Neuman et al., 2015). For example, HA and its pattern of fragmentation during wound repair contributes to the damage-associated molecular patterns (DAMPs) that trigger an inflammatory response (Schaefer, 2014). Blocking HA metabolism by deleting CD44 results in increased tissue HA accumulation in the lung following bleomycin-induced injury, leading to unremitting inflammation and ultimately tissue destruction (Jiang et al., 2011). As well, initiation and progression of cancer are profoundly affected by HA accumulation and metabolism, which is contributed to by mesenchymal cells within the peri-tumor stroma (Itano and Kimata, 2008; Kouvidi et al., 2014; Zhao et al., 2015). For example, elevated accumulation of HA in stroma is a poor prognostic factor in a number of cancers including oral squamous cell carcinoma, breast and prostate cancers (Tolg et al., 2014). CAFs, which can differentiate from mesenchymal progenitor cells, are heterogeneous, and one recently identified subpopulation produces high levels of HA that promotes motility and invasion of tumor cells. In addition, tumor microenvironments favor production of free radicals, which fragment HA into smaller polymers [29] thus replicating an environment that resembles that of wound. There is now increasing evidence that such microenvironments fuel tumor aggression. The HA binding functions of receptors expressed by stromal cells is therefore an important part of creating a pro-tumorigenic microenvironment.

We have shown previously that breast tumor cells are heterogeneous in their ability to bind to F-HA probes (Veiseh et al., 2014). Cell sorting based upon levels of F-HA binding show that this differential binding is associated with distinct phenotypic differences in tumor cells. Those that bind low levels of F-HA are poorly invasive but proliferate while those binding high levels of HA are highly invasive but proliferate slowly. Mesenchymal progenitor cells used in the present study were also heterogeneous in their ability to bind to F-HA, and this property may likewise be associated with differences in progenitor phenotypes (Caralla et al., 2012). At the least, F-HA binding may aid in enriching mesenchymal stem cells for therapeutic use particularly since isolation of these progenitors has been hampered by lack of specific surface markers. For example, mesenchymal stem cells in bone marrow aspirates can be separated from myeloid precursor cells based upon their relative ability to bind HA (Caralla et al., 2012).

In summary, here we have identified the HA receptors required for HA binding and internalization by a mesenchymal progenitor cell line. We show that depending upon the attachment status of these cells, different HA receptors dominate in carrying this function. Since mesenchymal progenitor cells traffic to sites of tissue injury which requires changes in their attachment status, and since HA metabolism affects many processes during wound repair and disease (e.g., cancer progression), our results may have clinical relevance for development of new mesenchymal-based therapies.

### Conflict of interest statement

The authors declare that the research was conducted in the absence of any commercial or financial relationships that could be construed as a potential conflict of interest.
